# Predictors of CT Morphologic Features to Identify Spread Through Air Spaces Preoperatively in Small-Sized Lung Adenocarcinoma

**DOI:** 10.3389/fonc.2020.548430

**Published:** 2021-01-11

**Authors:** Lin Qi, Ke Xue, Yongjun Cai, Jinjuan Lu, Xiaohu Li, Ming Li

**Affiliations:** ^1^ Department of Radiology, Huadong Hospital, Fudan University, Shanghai, China; ^2^ Department of Plastic and Reconstructive Surgery, Shanghai Ninth People’s Hospital, School of Medicine, Shanghai Jiao Tong University, Shanghai, China; ^3^ Department of Pathology, Huadong Hospital affiliated to Fudan University, Shanghai, China; ^4^ Department of Radiology, First Affiliated Hospital of Anhui Medical University, Hefei, China

**Keywords:** bronchial neoplasms, x-ray computed, tomography, adenocarcinoma of lung, spread through air spaces

## Abstract

**Objectives:**

This study aimed to explore the predictive CT features of spread through air spaces (STAS) in patients with small-sized lung adenocarcinoma.

**Methods:**

From January 2017 to May 2019, patients with confirmed pathology of small-sized lung adenocarcinoma (less than or equal to 2 cm) and who underwent surgery were retrospectively analyzed. The clinical, pathological, and surgical information and CT features were analyzed.

**Results:**

A total of 47 patients with STAS (males, 61.7%; mean age, 56 ± 8years) and 143 patients without STAS (males, 58%; mean age, 53 ± 11 years) were included. Pathologically, papillary, micropapillary, solid predominant subtypes, and vascular and pleural invasion were most commonly observed features in the STAS group. Radiologically, higher consolidation tumor ratio (CTR), presence of spiculation, satellites, ground glass ribbon sign, pleural attachment, and unclear tumor–lung interface were more commonly observed features in the STAS group. CTR, presence of ground glass ribbons and pleural connection, and absence of cystic airspaces were considered as stable predictors of STAS in multivariate logistic models. The receiver operating characteristic curve (ROC) analysis for predicting STAS demonstrated higher area under the curve (AUC) in the model that used CTR (0.760, 95% confidence interval, 0.69–0.83) for predicting STAS than in the model that used long diameter of entire lesion (0.640).

**Conclusions:**

CTR is the best CT sign for predicting STAS in small-sized lung adenocarcinoma. The ground glass ribbon is a newly found indicator and has the potential for predicting STAS.

## Highlights

1. Higher consolidation tumor ratio, presence of ground glass ribbons, satellites, pleural connection, and unclear tumor–lung interface are predictive CT features of STAS in small-sized adenocarcinoma.
2. During the follow-up of lung nodules, the ground glass ribbon sign is regarded as a new feature, although there is no increase in the entire size or solid component, arising suspicion for cancer with STAS.
3. In small-sized STAS-positive adenocarcinomas that are present near the interlobular fissure, the disseminated foci can spread along the congenital defect of the interlobar fissure towards the adjacent lung lobe.

## Introduction

The concept of spread through air spaces (STAS) is regarded as an additional pattern of invasion of lung cancer proposed by the World Health Organization classification of lung tumors in 2015. It consists of micropapillary clusters, solid nests, or single cells beyond the edge of the tumor invading into the air spaces surrounding the lung parenchyma (1). Validation studies on STAS considered it as a significant prognostic factor for distant and locoregional recurrence in patients undergoing limited resection, which decreased the overall survival in patients with lung cancers (2–5). STAS is an insidious invasive pattern that is not visible to pathologists on gross examination and to surgeons on external examination of tumor specimen at the time of surgery. So far, it remained unknown as to which imaging methods can be used for preoperative detection of it. For small-sized lung adenocarcinoma (less than or equal to 2 cm), limited resection can be usually performed, and its precise preoperative prediction of STAS on computed tomography (CT) before surgery remains crucial and challenging. This enables chest surgeons to change the treatment strategy from wedge resection to lobectomy, thereby reducing the recurrence rate. We hypothesize that some CT morphological predictors can predict STAS of small-sized lung adenocarcinoma before surgery, so as to avoid inappropriate sublobectomy of those patients. We performed comprehensive imaging and pathological statistical analysis to determine the imaging predictors predicting STAS in small-sized lung adenocarcinoma.

## Materials and Methods

This study has been approved by the institutional review board of Huadong Hospital, and patients’ informed consent was waived off due to retrospective nature of the study design.

### Study Population

**Figure 1:**
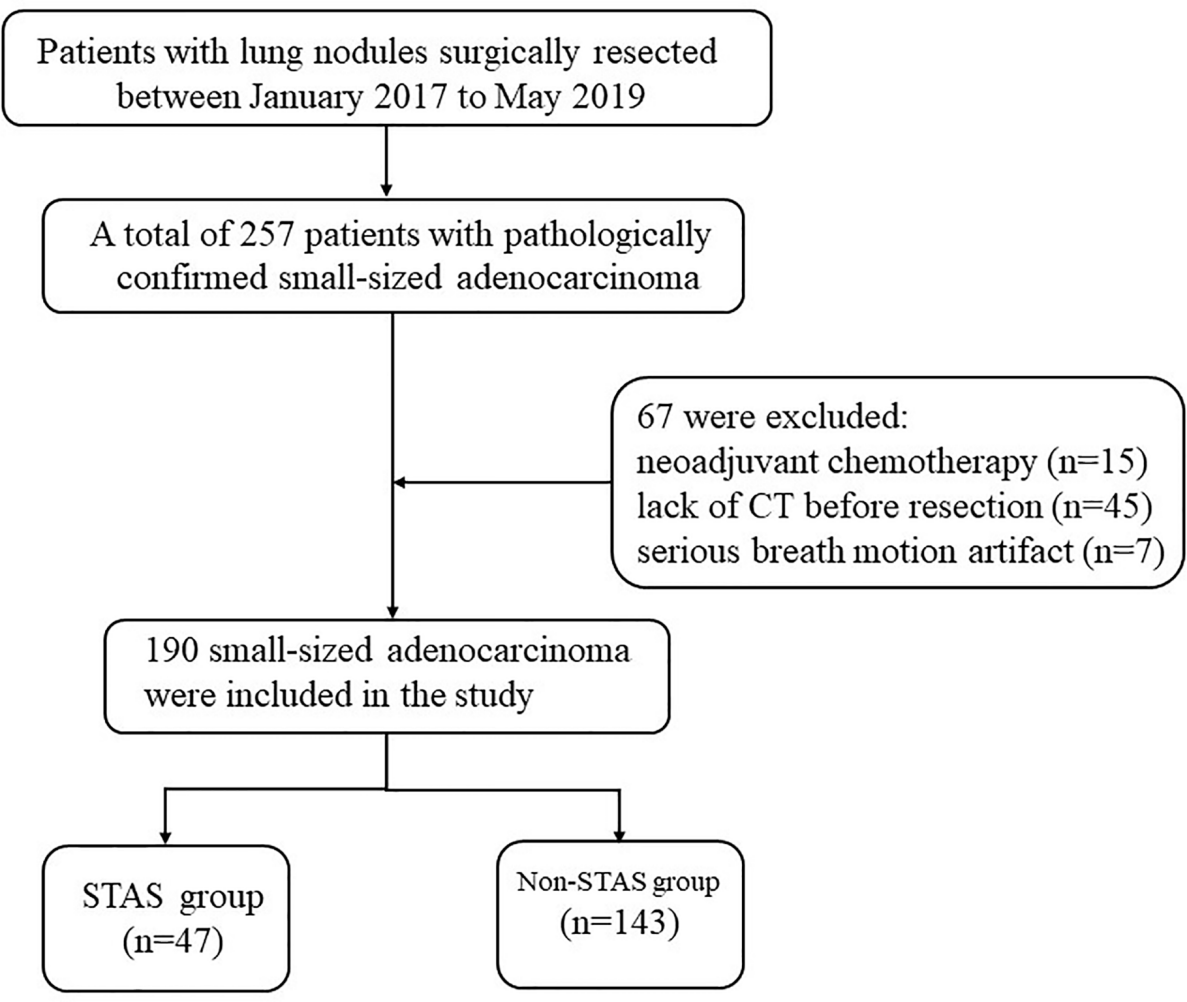
Flowchart of patient selection procedure. STAS, spread through air spaces; CT, computed tomography.

From January 2017 to May 2019, all consecutive patients at our institution were recruited, who underwent curative surgical resection and pathologically diagnosed with small-sized primary lung adenocarcinoma, which were staged T1–2 compliant with the eighth edition of the tumor, node, and metastasis (TNM) classification (6). A total of 257 patients with pathologically confirmed small-sized adenocarcinoma (equal or less than 2 cm in diameter) were included. The exclusion criteria were as follows: (1) patients who underwent preoperative neoadjuvant chemotherapy (n = 15); (2) with a time interval more than 3 months between the last CT examination and surgical resection, or who did not undergo CT examination in our hospital (n = 45); and (3) with serious breathing artifacts on CT image (n = 7). The flowchart of patients’ selection was shown in **Figure 1**.

The information regarding the following parameters were collected from the database: (1) patient characteristics (such as age, gender, smoking status, tumor markers); (2) surgical methods (pneumonectomy, lobectomy, segmentectomy, or wedge resection); (3) pathological findings (composition, lymph node metastasis, pleural/vessel invasion, STAS); (4) epidermal growth factor receptor (EGFR) mutations; and (5) CT characteristics.

### Pathological Analysis

Pathological diagnosis and categorization of adenocarcinoma were done based on the 2011 edition of pulmonary adenocarcinoma classification (7). Surgically resected specimens were fixed in 10% formalin, placed in paraffin to block, sectioned into thin sections, and then stained with hematoxylin and eosin. Pathological analysis was performed by a pathologist with 32 years of experience (Li Xiao). STAS was considered to exist when the micropapillary clusters, solid nests, or single cells spread within the air spaces beyond the edge of the main tumor according to the 2015 WHO classification (8). Tumor cells that spread through the mucus were distinguished from STAS, and the edge of the adenocarcinoma with mucinous characteristics was defined as alveolar space filled with mucin. EGFR mutation was evaluated in all participants.

### CT Acquisition and Analysis

Chest CT scans were obtained with 64-slice Discovery CT750 HD (GE Healthcare) and 64-slice GE Light speed VCT using similar protocols, which were as follows: 1.25-mm slice thickness with a 1.25-mm reconstruction interval; pitch of 0.984; tube voltage, 120 kVp; tube current, 250 mAs; and bone reconstruction kernel. All images were reviewed and measured with a standard lung window (window width, 1,500 HU; window level, −500 HU) and a mediastinal window (window width, 350 HU; window level, 50 HU). All lesions were measured on the maximum plane of axial images.

The size of the entire lesion was defined as the average of long and short axial diameters, and all the measurements were rounded to the nearest millimeter according to the 2017 guidelines for management of incidental pulmonary nodules from the Fleischner Society (9). All lesions with long axial diameters were measured on the maximum transverse reconstructed images. For mixed ground-glass nodules (mGGNs), the size of the solid components was measured using the same method as that of a mediastinal window. The maximum long diameter was used to define the TNM stage. The consolidation tumor ratio (CTR) was defined as the proportion of maximum consolidation diameter divided by the maximum tumor diameter.

The morphological characteristics of the nodules such as nodule density (solid, part solid, or pure ground glass), location, shape (round or oval, irregular), vascular change (normal, convergent or dilated), and cystic air spaces were included. Air spaces were defined as containing congenital cysts, emphysematous bullae, and bronchiectasis airways, which in turn were comprised of bubble-like lucencies and air bronchogram simultaneously.

The edge features of the lesions were as follows: (1) ground glass ribbon sign is a CT finding with a band-shaped ground glass opacity and blurred edge, which was emitted from the edge of the nodule and extended to the adjacent lung. If the lesion was present close to the visceral pleura, then it reaches the adjacent visceral pleura, without pleura thickening, pulling, or indentation; (2) tumor–lung interface: clear or unclear; (3) lobulation: is defined as a wavy or scalloped configuration of the edge of the nodule; and (4) spiculation sign: is defined as the presence of strands with soft tissue density that extends from the margin of the nodule into the lung.

Nodule-pleural types: (1) no connection, (2) attachment, with or without indentation, and (3) closeness through ground glass ribbon or spiculation, with or without traction. Morphological analysis was performed by two radiologists with nine and 15 years of experience in chest radiologic diagnosis (Lin Qi and Ming Li), and were blinded to the results of the pathological results. Any disagreements between them were resolved by reaching a consensus.

### Statistical Analysis

Statistical analysis was carried out by SPSS 22.0 software and GraphPad Prism. The values were described as means ± standard deviation (SD) or sample rate. The normality of variables was tested using Shapiro–Wilk test. For categorical variables, the data between the two groups were compared by Pearson’s chi-squared test and Fisher’s exact test. For continuous variables, the data were compared by unpaired t test or Mann–Whitney U test. Binary logistic analysis for multivariate regression analysis was performed to identify independent predictors of adenocarcinoma with STAS. Variables with p values less than 0.1 by pairwise comparison were included in multivariate analysis. ROC analysis and Youden index were used to determine the cutoff value of CTR for predicting adenocarcinomas with STAS. For binary logistic analysis and ROC analysis, the nodular density of the lesions was divided into two types: solid nodules and subsolid nodules; the satellite lesions into present and absent types; and nodule-pleural relationships were divided into connection and non-connection types. The AUCs of the predictive CT features were compared by DeLong test. A *p* value of less than 0.05 was considered to be significant.

## Results

### Demographics, Surgical, and Pathological Data

Demographics, surgical strategies, and pathological data were presented in **Table 1**. One hundred and ninety patients with small-sized lung adenocarcinoma were finally enrolled in our study, which included 47 patients with STAS and 143 patients without STAS. The mean age of the patients was 55 ± 14 years and included 112 males and 78 females. Fifty-six participants were heavy smokers (>30 packs/year and quit smoking within the past 15 years). Seventy-one (37.4%) participants underwent sublobar resection, and 119 (62.6%) accepted lobectomy or pneumonectomy. There were no significant differences between STAS and non-STAS groups with regard to age, sex, heavy smokers, and surgery strategy. Significant differences between the two groups were observed in predominant histologic subtypes, where acinar and lepidic are the most common predominant subtypes in the non-STAS group, while papillary, micropapillary, and solid subtypes were more commonly observed in the STAS group. There was no significant difference observed in cribriform subtype between the two groups. Vascular (*p* = 0.001) and pleural invasion (*p* < 0.0001) were the most commonly observed in adenocarcinomas with STAS patients. No significant difference was present in EGFR mutations.

**Table 1:** Demographics, pathological analysis, and types of surgery of participants with pathologically T1a and T1b lung adenocarcinoma.

	Total (n = 190)	STAS (n = 47)	Non-STAS (n = 143)	t or z value	p
Age (years)	55 ± 14	56 ± 8	53 ± 11	1.778	0.100
Male, n [%]	112 [58.9]	29 [61.7]	83 [58]	0.443	0.658
Heavy smoke, n [%]	56 [29.5]	16 [34.0]	37 [25.9]	1.083	0.279
Surgery				1.933	0.05
Sublobar resection, n [%]	71 [37.4]	12 [25.6]	59 [41.3]		
Lobectomy or pneumonectomy, n [%]	119 [62.6]	35 [74.5]	84 [58.7]		
Pathology					
Predominant histologic subtypes					
Lepidic, n [%]	30 [15.8]	0	30 [21.0]	–	0.0001^***^
Acinar, n [%]	87 [45.8]	5 [10.6]	82 [57.3]	–	<0.0001^****^
Papillary or micropapillary, n [%]	38 [25.9]	27 [57.4]	11 [7.7]	7.398	<0.0001^****^
Solid, n [%]	26 [13.7]	12 [25.5]	14 [9.8]	2.724	0.006^**^
Cribriform, n [%]	9 [4.7]	3 [6.4]	6 [4.2]	–	0.692
Vascular invasion (+), n [%]	26 [13.7]	12 [25.6]	11 [7.7]	3.253	0.001^**^
Pleural invasion (+), n [%]	31 [16.3]	19 [40.4]	17 [11.9]	4.331	<0.0001^****^
EGFR mutation (+), n [%]	78 [41.1]	12 [25.5]	66 [46.2]	0.493	0.013*

STAS, spread through air spaces; EGFR, epidermal growth factor receptor. Significant level marks: p < 0.05 *, p < 0.01 **, p < 0.001 ***, p < 0.0001 ****.

### CT Characteristics of Two Groups

**Figure 2:**
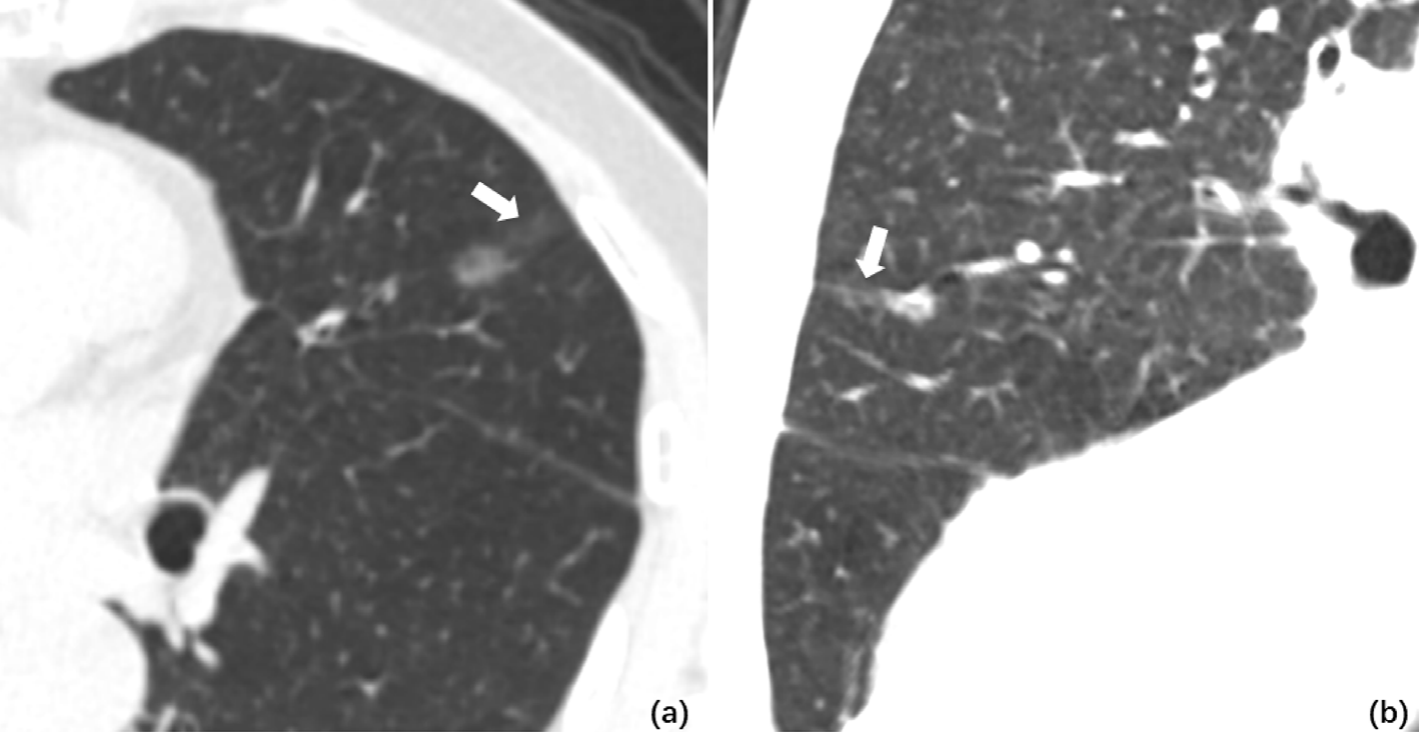
**(A)** Axial CT image of pGGO performed STAS pathologically. A ground glass ribbon sign was observed on the margin of the lesion and extended to the adjacent costal parietal pleura (white arrow), which was defined as a CT finding of a band-shaped ground glass opacity with blurred edge that emits from the edge of the nodule and extends into the adjacent lung **(B)**. Multi-plane reconstruction with ground glass ribbon sign as the long axis. The shape of ribbon sign was displayed more clearly after adjusting the window width and level.

CT characteristics of all lesions in STAS and non-STAS groups were shown in **Table 2**. No significant differences were shown in the long axial diameter of entire tumor and solid component, but a statistically significant difference was observed in CTR between the STAS and non-STAS groups (0.9 ± 0.2 *vs.* 0.6 ± 0.4, *p* = 0.0001). Only one case (1/47, 2.1%) in the STAS group showed pure ground glass density (**Figure 2**), while 32 cases (32/143, 22.4%) in the non-STAS had pure ground glass nodules (pGGNs, p = 0.0007). The number of SNs in the STAS group was more than that in non-STAS group (61.7 *vs.* 28.7%, *p* < 0.0001). The number of mGGNs showed no significant differences between the two groups.

**Table 2:** CT characteristics of small-sized adenocarcinoma in STAS+ and STAS- groups.

	Total (n=190)	STAS (n=47)	Non-STAS (n=143)	t, F, or z value	p value
Measurements					
LD _entire_ (mm)	15 ± 4	16 ± 4	14 ± 3	4.025	0.055
LD _solid_ (mm)	10 ± 6	15 ± 5	9 ± 6	2.408	0.122
CTR	0.7 ± 0.4	0.9 ± 0.2	0.6 ± 0.4	23.868	0.0001***
Nodule density					
pGGNs	33 [17.4]	1 [2.1]	32 [22.4]	–	0.0007***
mGGNs	87 [45.8]	17 [36.2]	70 [49.0]	1.526	0.127
SNs	70 [36.8]	29 [61.7]	41 [28.7]	4.073	<0.0001****
Location (lobe)				0.588	0.557
Upper lobes	94 [49.5]	25 [53.2]	69 [48.3]		
Non-upper lobes	76 [40]	22 [46.8]	74 [51.7]		
Location (field)				0.326	0.745
Central	69 [36.3]	18 [38.3]	51 [35.7]		
Peripheral	121 63.7]	29 [61.7]	92 [64.3]		
Shape				1.849	0.064
Round or oval	95 [50]	29 [61.7]	66 [46.2]		
Irregular	95 [50]	18 [38.3]	77 [53.8]		
Vascular change				0.602	0.547
normal	106 [55.8]	28 [59.6]	78 [54.5]		
convergent	84 [44.2]	19 [40.4]	65 [45.5]		
Cystic airspaces, n [%]	73 [38.4]	11 [23.4]	62 [43.4]	2.440	0.015*
Edge features					
Ground glass ribbon sign	34 [17.9]	22 [46.8]	12 [8.4]	5.961	<0.0001****
Unclear tumor-lung interface	38 [20]	15 [31.9]	23 [16.1]	2.354	0.019*
Lobulation	70 [36.8]	16 [34.0]	54 [37.8]	0.459	0.647
Spiculation	92 [48.4]	34 [72.3]	58 [40.6]	3.782	0.0002***
Satellite lesions					
Absent	171 [90]	31 [66.0]	140 [97.9]	–	<0.0001****
Ground glass satellites	9 [4.7]	9 [19.4]	0	–	<0.0001****
Solid satellites	10 [5.3]	7 [14.9]	3 [2.1]	–	0.003**
Nodule-pleural types					
No connection	93 [48.9]	11 [23.4]	82 [57.3]	4.038	<0.0001****
Closeness	69 [36.3]	21 [44.7]	48 [33.6]	1.369	0.169
Attachment	28 [14.7]	15 [31.9]	13 [9.1]	3.83	0.0001***

LD _entire_, long diameter of entire tumor; LD _solid_, long diameter of solid component; CTR, consolidation tumor ratio; STAS, spread through air spaces; pGGNs, pure ground glass nodules; mGGNs, mixted ground glass nodules; SNs, solid nodules. Significant level marks: p < 0.05 *, p < 0.01 **, p < 0.001 ***, p < 0.0001 ****.

**Figure 3:**
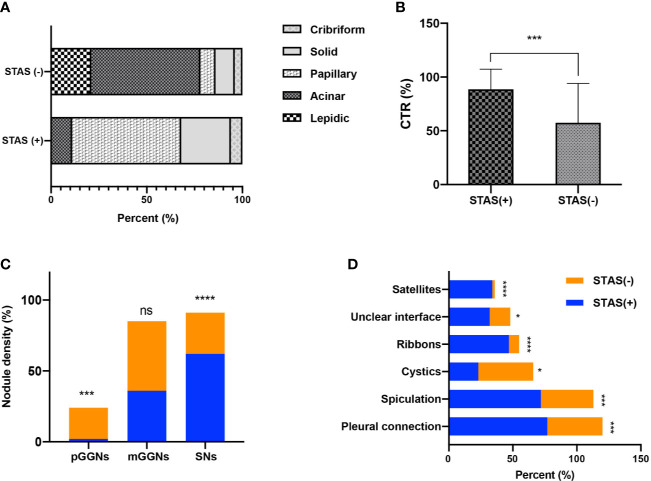
Bar graph shows the comparison of predominant subtypes between STAS-positive and STAS-negative groups **(A)**; there is statistically significant difference in CTR between the two groups **(B)**; the number of SNs in STAS group is more than that in non-STAS group **(C)**; presence of spiculation, satellites, and ground glass ribbon sign, pleural attachment, and unclear tumor–lung interface was more common in STAS group **(D)**. *P<0.05; **P<0.01; ***P<0.001; ****P<0.0001.

There were no significant differences regarding lobe location, shape, lobulation, and vascular changes. Spiculation was more frequently observed in the STAS groups (72.3 *vs.* 40.6%, *p* = 0.0001). The ground glass ribbon sign and the unclear tumor–lung interface were more commonly observed in the STAS group than that in non-STAS group (68 *vs.* 5.6%, p < 0.0001; 31.9 *vs.* 16.1%, p = 0.019). In the STAS group, there were more GGNs and solid satellites around the tumor than those in the non-STAS group (19.4 *vs.* 0, p < 0.0001; 14.9 *vs.* 2.1%, p = 0.003). More STAS lesions had attachment to the visceral pleura than non-STAS lesions (31.9 *vs.* 9.1%, p = 0.0001) (**Figure 3**).

### CT Characteristics for Predicting STAS

**Figure 4:**
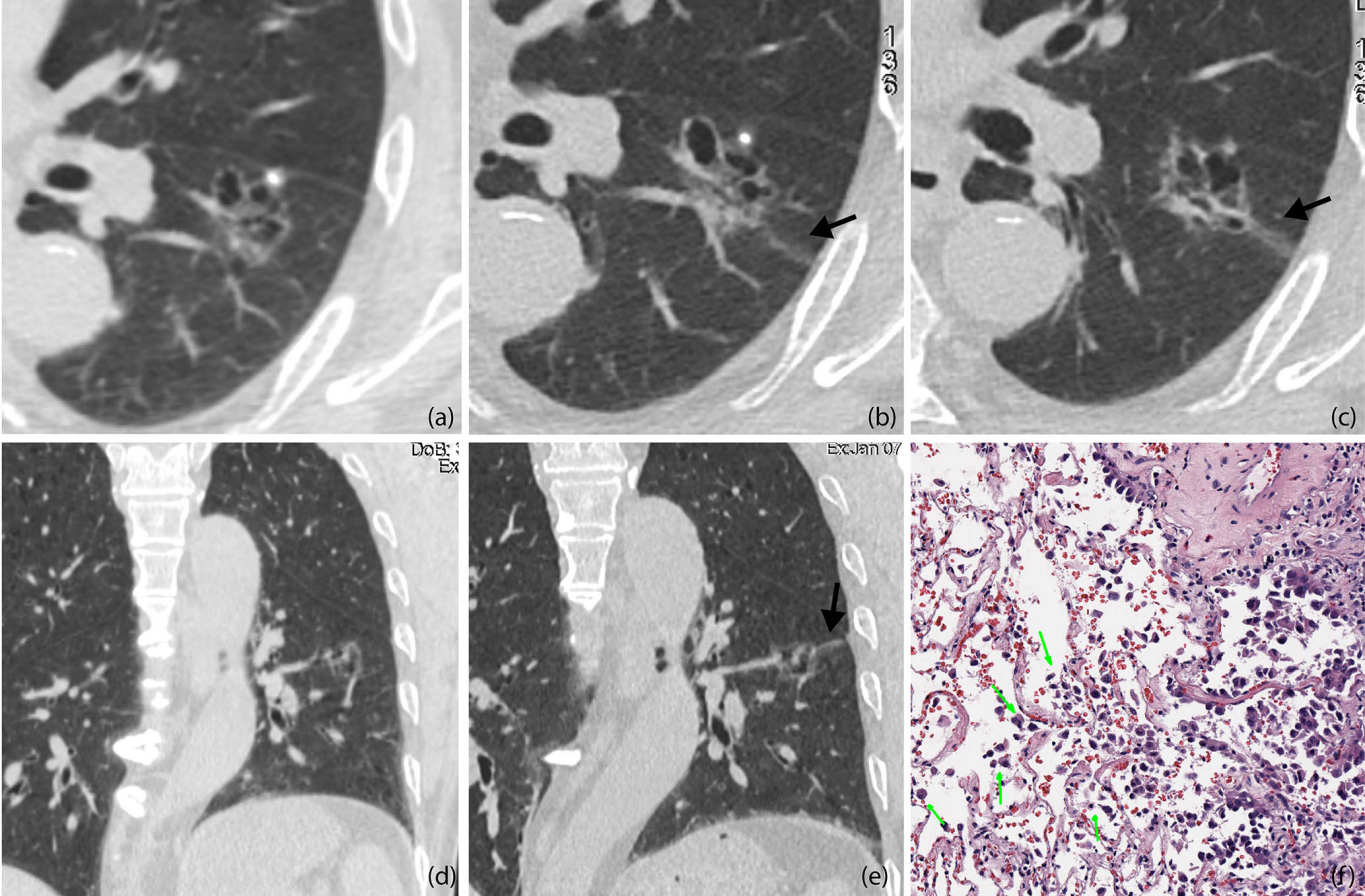
**(A, D)** Baseline axial **(A)** and coronal **(D)** CT images of STAS-positive adenocarcinoma in a 51-year-old man. An irregular ground glass nodule with multiple cystic cavities was detected with clear edges, so he was recommended 6–12 months follow-up **(B, C, E)**. Axial CT images on the 8-month follow-up **(B, C)**. The entire size of the lesion did not change, but some cysts were larger than before, and a ground glass ribbon sign was newly found on the edge of the lesion (black arrow), which was defined as a CT finding of a band-shaped ground glass opacity with blurred edge that emits from the edge of the nodule and extends into the adjacent lung **(E)**. Coronal reconstructed image clearly shows the ribbon sign, stretching to the adjacent visceral pleural (black arrow) **(F)**. The patient was recommended lung lobe resection and was confirmed invasive adenocarcinoma with STAS pathologically. Photomicrograph shows single cell pattern STAS consisting of scattered discohesive single cells (green arrow).

The imaging features of STAS were evaluated by conducting multivariate logistic analysis (**Table 3**) and receiver operating characteristic curve analysis. Variables with *p* values of less than 0.10 in univariate analysis were included in multivariate logistic analysis, which included CTR, shape, spiculation, cystic airspaces, ground glass ribbons, tumor–lung interface, nodule density, satellite lesions, and pleural connection (**Figure 4**). CTR and nodule density showed significant correlation, so CTR was separately listed by establishing two regression models. In model 1, the presence of cystic airspaces, ground glass ribbon sign, unclear tumor–lung interface, pleural connection, and CTR were considered as predictive factors in STAS patients (p < 0.05). In model 2 that included CTR and nodule density simultaneously, the predictive factors were similar to the results of model 1.

**Table 3:** Multivariable Logistic Analysis of radiologic predictors of adenocarcinoma with STAS.

	Model 1			Model 2		
	OR	95% CI	*p*-value	OR	95% CI	*p*-value
Nodule shapes	2.155	0.768–6.048	0.145	0.509	1.18–1.45	0.206
Spiculation	0.977	0.242–3.947	0.974	0.509	2.19–3.45	0.206
Cystic airspaces	13.781	3.751–50.63	0.001	0.062	0.02–0.25	0.001
Ground glass ribbon sign	0.114	0.029–0.454	0.002	8.468	2.03–35.32	0.003
Boundary	0.243	0.07–0.842	0.026	4.871	1.31–18.12	0.018
Satellite lesions	0.585	0.176–1.947	0.382	3.263	0.45–23.48	0.24
Pleural connection	0.183	0.059–0.563	0.003	5.829	1.80–18.93	0.003
Nodule density	–	–	–	0.146	0.00–2.34	0.086
CTR	0.01	0.001–0.173	0.001	0.003	0.00–0.10	0.001

CTR, consolidation tumor ratio; CI, confidence interval; OR, odds ratio.

**Figure 5:**
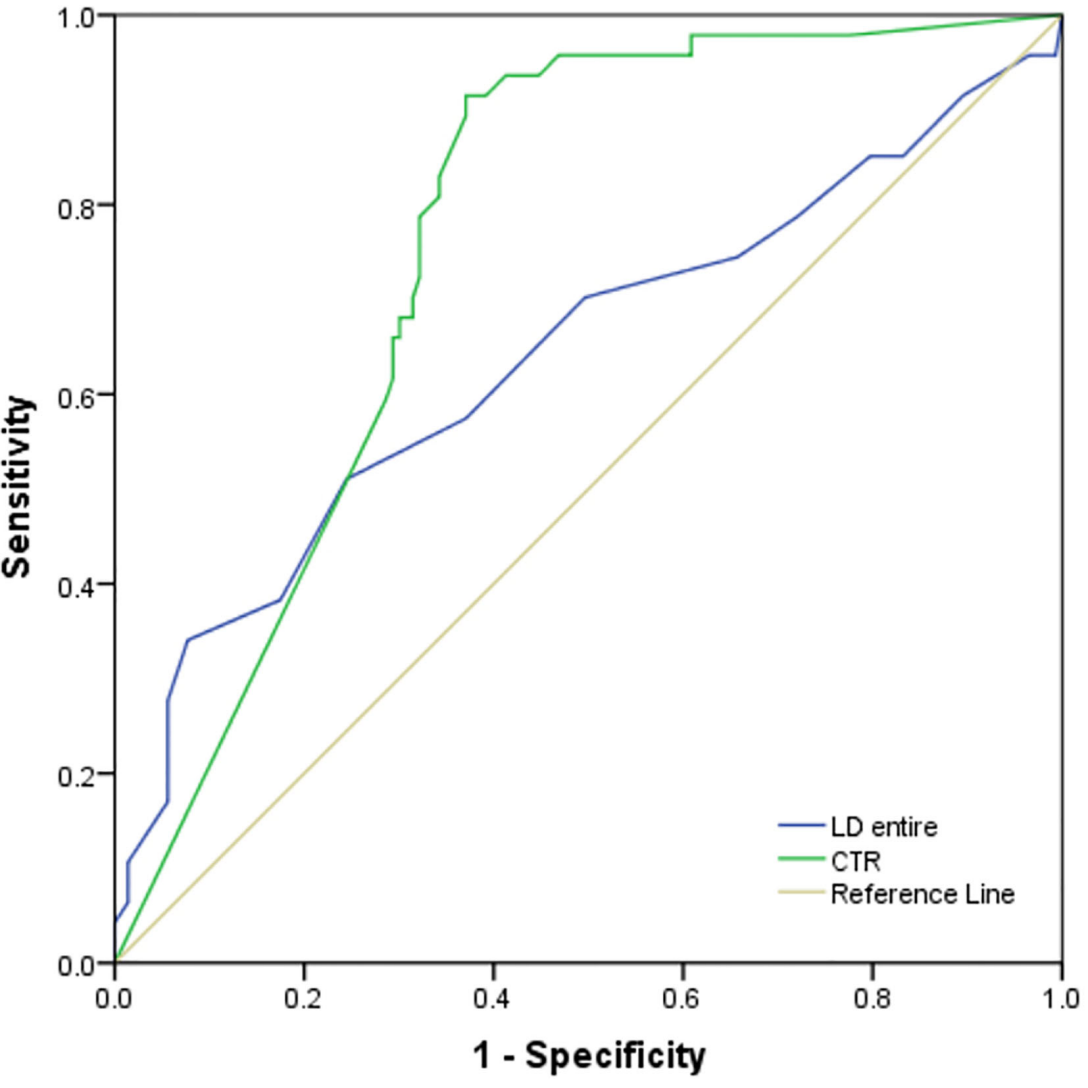
The ROC analysis for predicting STAS demonstrated higher area under the curve (AUC) in the model that used CTR (0.760, 95% confidence interval, 0.69–0.83) for predicting STAS than in the model that used long diameter of entire lesion (0.640).

The ROC analysis for predicting STAS demonstrated higher area under the curve (AUC) in the model that used CTR (0.760, 95% confidence interval, 0.69–0.83) for predicting STAS than in the model that used long diameter of entire lesion (0.640). (**Figure 5**). There was no statistical significance in the differences between AUCs of CTR and diameter of entire lesion (p < 0.05).

## Discussion

STAS is recently recognized as an additional pattern of tumor invasion in lung adenocarcinoma and is considered as a major risk factor of recurrence in early stage lung adenocarcinoma patients when treated with limited resection (2, 3, 10–13). Kadota K et al. (2) have reported that the risk of recurrence, which included the 5-year cumulative incidence of recurrence, distant and locoregional recurrence, was significantly higher in patients with STAS-positive small-sized lung adenocarcinoma when compared to those with STAS-negative tumors. Therefore, retrospective analysis was conducted to those pathologically proved small-sized lung adenocarcinoma with and without STAS, exploring whether CT features could assist in predicting STAS before undergoing surgical procedures to decide tumor margin in conditions of limited resection and guide the choice of postoperative treatment strategies (8, 14, 15).

Our study confirmed that STAS-positive small-sized lung adenocarcinomas had distinct morphologic, pathologic, and genetic characteristics, and CTR was an important imaging predictor of STAS, which was similar to the results of previous studies (4, 10, 11, 16, 17). Pathologically, the predominant subtypes of STAS positive adenocarcinoma included papillary, micropapillary and solid, while that of STAS negative tumors included lepidic and acinar. The vascular and pleural invasion and EGFR mutation were regarded as the more common features in STAS positive adenocarcinoma.

Recent studies confirmed that direct signs of STAS are far beyond the spatial resolution of state-of-art CT scanner, even on high-resolution CT images, which demonstrating that the prediction of STAS on CT images should be performed by indirect signs rather than direct visualization (18). Kim et al. reported a larger series of STAS+ adenocarcinomas (n = 94) containing different tumor sizes and pathological stages and suggested that the percentage of solid component was an independent predictor of STAS and a cut-off value of 90% with a sensitivity of 89.2% and a specificity of 60.3%. Our study focuses on the morphological features of small-sized lung adenocarcinoma positive for STAS. Therefore, there are some similarities as well as differences in conclusions due to the different research groups. Similar to Kim et al., we found that CTR (equivalent to percentage of solid component in Kim’s study) was the best predictive CT feature with a cut-off value of 83% and with a sensitivity of 91.5%, a specificity of 62.9%. The diagnostic efficacy of CTR in the two studies is similar, indicating that even if the study populations are different, CTR shows stable prediction performance in predicting STAS. In our study, no differences were found in maximum diameters of the entire lesion and solid component between tumors positive for STAS and that negative for STAS, while Kim et al. reported that the maximum diameter of solid component was greater in STAS-positive adenocarcinoma. The reason might be that our study limited the maximum diameter of the included lesions (less than 2 cm in diameter), while Kim’s study did not. In our cohort, 61.7% of STAS-positive tumors were solid nodules (CTR = 1), while 71.3% of STAS-negative lesions were non-solid lesions, indicating that solid small-sized tumors were more likely accompanied with STAS. This suggested the requirement of partial resection with enlarged margin or lobe resection.

Several studies (19–21) showed that satellite centrilobular nodules, branching opacities, typically with ill-defined margins and ground glass attenuation, are discriminatory CT characteristics that are suggestive of macroscopic tumor spread through airways. Similar to those studies, our study confirmed that several signs on CT images could predict STAS in small-sized primary lung adenocarcinoma, including presence of spiculation, satellites, ground glass ribbon sign, pleural attachment, and unclear tumor–lung interface. Among these features, some edge characteristics, such as ground glass ribbon sign, spiculation, and ground glass satellites demonstrated strong discriminative power than the remaining ones. Ground glass ribbon sign is the newly proposed CT feature in our study for indicating STAS and was defined as a CT finding of a band-shaped ground glass opacity with blurred edge that emits from the edge of the nodule and extends into the adjacent lung. Pathologically, this sign may be related to decreased air spaces in the distal part of the alveoli that is caused by the obstruction of the surrounding lung parenchyma beyond the tumor, or the obstruction of terminal bronchiolar. The predominant composition of STAS-negative small-sized adenocarcinoma was usually considered to be the lepidic subtype, and it was usually well-defined, without terminal bronchiolar obstruction and corresponding ground glass ribbon sign. While in STAS-positive adenocarcinoma, micropapillary clusters and solid nests surrounding the air spaces could block the marginal alveolar cavities, leading to a decrease in the air content of the distal alveolar cavities. Several studies reported that STAS does not occur in pGGNs (10, 18–21).

**Figure 6:**
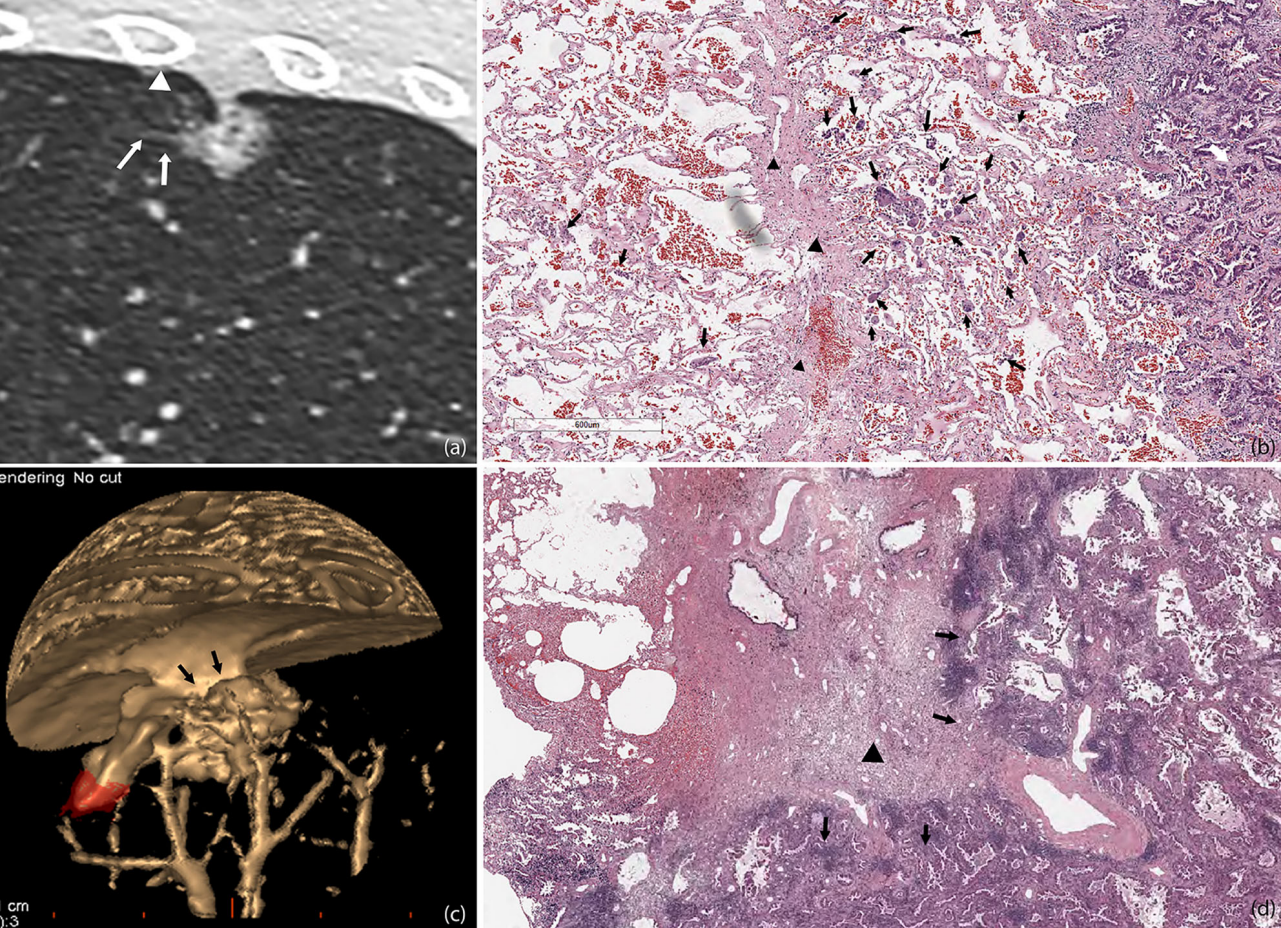
STAS in a 45-year-old man with micropapillary predominant subtype of invasive adenocarcinoma **(A)**. Multiplanar reconstructed CT image (width, 1,500 HU; level, −600 HU) shows a ground glass-density spiculation extending into the adjacent lungs (arrow), and several ground glass-density satellite foci with a diameter of 1–2 mm were observed at the edge of the lesion (white triangle) **(B)**. Photomicrograph shows multiple solid nests clusters alveolar disseminations at the edge of one side of the lesion (arrow), spreading through the interlobular fissure (black triangle) to the alveolar cavities of the adjacent pulmonary lobe **(C)**. Volume render reconstructed image shows visceral pleural indentation **(D)**. Photomicrograph shows visceral pleural invasion, indentation, and thickening.

In our study, only one patient with pGGO was pathologically confirmed to have STAS, and a ground glass ribbon sign was observed on the margin of the lesion, which was extended to the adjacent costal parietal pleura. The reason for this might be that resection was more likely performed for partly solid or solid nodules, and most of the pGGOs were recommended to follow-up until they become partially solid nodules before undergoing resection. We alluded that in the setting of GGO adenocarcinoma the Ribbon sign might be an early marker of STAS before a solid component develops. This might be potentially used as an indication for early resection of slowly growing GGOs. However, future multicenter validation studies are needed to consolidate our findings. Masai et al. (3) demonstrated that STAS and tumor margins smaller than 1 cm are significant risk factors for local recurrence in early stage lung cancer after limited resection. In our study, we observed on pathological sections that tumor cells of STAS-positive small-sized adenocarcinoma can spread to the adjacent lung lobe through the congenital pores of the interlobular fissure (**Figure 6**). It can be shown that pathological STAS is one of the risk factors for metastasis of early adenocarcinoma after local pneumonectomy.

Our study has several limitations. Firstly, this was a retrospective single-center study, and the sample size included was small. So, comparative studies with a large number of pathological and CT images are needed to confirm these assumptions. Secondly, we only included adenocarcinomas in our study. Different histologic types of lung cancer and some inflammatory granulomas that are easily confused with lung cancer should also be included in the future studies. Thirdly, due to a shorter postoperative follow-up time, our study did not report the prognostic information of the two groups. Future research will assess the prognostic impact of STAS and local recurrence in stage I lung adenocarcinoma.

In conclusion, CTR is the most robust CT sign for predicting STAS in small-sized lung adenocarcinoma. Other CT features also have good diagnostic efficacy factors including spiculation, the ground glass ribbon sign, pleural connection, and satellites. Among them, the ground glass ribbon is a newly found indicator and has the potential for predicting STAS.

## Data Availability Statement

The original contributions presented in the study are included in the article/supplementary materials. Further inquiries can be directed to the corresponding author.

## Ethics Statement

The studies involving human participants were reviewed and approved by the institutional review board of Huadong Hospital, and patients’ informed consent was waived off due to retrospective nature of the study design. Written informed consent for participation was not required for this study in accordance with the national legislation and the institutional requirements.

## Author Contributions

LQ and KX: manuscript writing and data analysis. YC: pathology diagnosis. JL: data organizing and analysis. XL: manuscript revision. ML: research decision. All authors contributed to the article and approved the submitted version.

## Funding

This study was supported by the National Natural Science Foundation of China (61976238); National Natural Science Foundation of China (82071897); Research Fund of Huadong Hospital (2019lc008); Shanghai Municipal Health Commission (20204Y0299); Medical Imaging Key Program of Wise Information Technology of 120, Health Commission of Shanghai 2018ZHYL0103 (ML), “Future Star” of famous doctors’ training plan of Fudan University.

## Conflict of Interest

The authors declare that the research was conducted in the absence of any commercial or financial relationships that could be construed as a potential conflict of interest.

## Abbreviations

CT, Computed tomography; STAS, Spread through air spaces; CTR, Consolidation tumor ratio; AUC, Area under the curve; ROC Receiver operating characteristic; mGGNs, Mixed ground-glass nodules; EGFR, Epidermal growth factor receptor; pGGNs, Pure ground-glass nodules.
